# Laxatives or methylnaltrexone for the management of constipation in palliative care patients

**DOI:** 10.1590/S1516-31802011000400014

**Published:** 2011-05-05

## Abstract

**BACKGROUND:**

Constipation is common in palliative care; it can generate considerable suffering due to the unpleasant physical symptoms. In the first Cochrane Review on effectiveness of laxatives for the management of constipation in palliative care patients, published in 2006, no conclusions could be drawn because of the limited number of evaluations. This article describes the first update of this review.

**OBJECTIVE:**

To determine the effectiveness of laxatives or methylnaltrexone for the management of constipation in palliative care patients.

**CRITERIA FOR CONSIDERING STUDIES FOR THIS REVIEW:**

We searched databases including MEDLINE and CENTRAL (The Cochrane Library) in 2005 and in the update to August 2010.

**SELECTION CRITERIA:**

Randomized controlled trials (RCTs) evaluating laxatives for constipation in palliative care patients. In the update we also included RCTs on subcutaneous methylnaltrexone; an opioid-receptor antagonist that is now licensed for the treatment of opioid-induced constipation in palliative care when response to usual laxative therapy is insufficient.

**DATA COLLECTION AND ANALYSIS:**

Two authors assessed trial quality and extracted data. The appropriateness of combining data from the studies depended upon clinical and outcome measure homogeneity.

**MAIN RESULTS:**

We included seven studies involving 616 participants; all under-reported methodological features. In four studies the laxatives lactulose, senna, co-danthramer, misrakasneham, and magnesium hydroxide with liquid paraffin were evaluated. In three methylnaltrexone.

**AUTHORS’ CONCLUSIONS:**

The 2010 update found evidence on laxatives for management of constipation remains limited due to insufficient RCTs. However, the conclusions of this update have changed since the original review publication in that it now includes evidence on methylnaltrexone. Here it found that subcutaneous methylnaltrexone is effective in inducing laxation in palliative care patients with opioid-induced constipation and where conventional laxatives have failed. However, the safety of this product is not fully evaluated. Large, rigorous, independent trials are needed.

Scientific Director, National Academy of Palliative Care (Academia Nacional de Cuidados Paliativos; ANCP), São Paulo, Brazil.

**This is the abstract of a Cochrane Review published in the Cochrane Database of Systematic Reviews (CDSR) 2011, Issue 2, DOI:** DOI: 10.1002/14651858.CD003448.pub4 (www.thecochranelibrary.com). For full citation and authors details see reference 1.

**For Latin America and the Caribbean, the full text is freely available from:** http://cochrane.bvsalud.org/cochrane/show.php?db=reviews&mfn=1839&id=CD003448&lang=pt&dblang=&lib=COC&print=yes

**This section was edited under the responsibility of the Brazilian Cochrane Center**

The independent commentary was written by Ricardo Tavares de Carvalho.

## COMMENTS

Opioid-induced constipation is one of the most common issues in palliative care practice. However, rational use of laxatives tends to be effective treatment for most cases. Besides this, maintaining good hydration and avoiding remaining immobile, if possible, are strongly recommended.

Methylnaltrexone is an opioid-receptor antagonist recently approved by the United States Food and Drug Administration (FDA) and has shown promising results in this group of patients. However, its high cost makes rational indication necessary, directed specifically towards cases that are resistant to other laxatives, with careful use and even with use in association.

There is still a lack of evidence from randomized controlled studies (RCTs). New RCTs would make it possible to better understand the risks, benefits and security issues of this new drug.
